# All-Climate Aluminum-Ion Batteries Based on Binder-Free MOF-Derived FeS_2_@C/CNT Cathode

**DOI:** 10.1007/s40820-021-00682-8

**Published:** 2021-07-23

**Authors:** Yuxiang Hu, Hongjiao Huang, Deshuang Yu, Xinyi Wang, Linlin Li, Han Hu, Xiaobo Zhu, Shengjie Peng, Lianzhou Wang

**Affiliations:** 1 Nanomaterials Centre, School of Chemical Engineering and Australian Institute for Bioengineering and Nanotechnology, The University of Queensland, Brisbane, Queensland 4072 Australia; 2 Jiangsu Key Laboratory of Electrochemical Energy Storage Technologies, College of Materials Science and Technology, Nanjing University of Aeronautics and Astronautics, Nanjing, 211106 People’s Republic of China; 3 State Key Laboratory of Heavy Oil Processing, College of Chemical Engineering, University of Petroleum (East China), Qingdao, 266580 People’s Republic of China

**Keywords:** Aluminum-ion battery, All-climate battery, Iron sulfide, Binder-free, High rate capacity

## Abstract

Aluminum-ion batteries (AIBs) are promising next-generation batteries systems because of their features of low cost and abundant aluminum resource. However, the inferior rate capacity and poor all-climate performance, especially the decayed capacity under low temperature, are still critical challenges toward high-specific-capacity AIBs. Herein, we report a binder-free and freestanding metal–organic framework-derived FeS_2_@C/carbon nanotube (FeS_2_@C/CNT) as a novel all-climate cathode in AIBs working under a wide temperature window between −25 and 50 °C with exceptional flexibility. The resultant cathode not only drastically suppresses the side reaction and volumetric expansion with high capacity and long-term stability but also greatly enhances the kinetic process in AIBs with remarkable rate capacity (above 151 mAh g^−1^ at 2 A g^−1^) at room temperature. More importantly, to break the bottleneck of the inherently low capacity in graphitic material-based all-climate AIBs, the new hierarchical conductive composite FeS_2_@C/CNT highly promotes the all-climate performance and delivers as high as 117 mAh g^−1^ capacity even under −25 °C. The well-designed metal sulfide electrode with remarkable performance paves a new way toward all-climate and flexible AIBs.

**Supplementary Information:**

The online version contains supplementary material available at 10.1007/s40820-021-00682-8.

## Highlights

- A binder-free and freestanding all-climate cathode FeS_2_@C/CNT in aluminum-ion batteries working from −25 to 50 °C with exceptional flexibility, enhanced capacity retention (above 117 mAh g^−1^) and rate capacity even at a low temperature of −25 °C.
- High rate capacity (above 151 mAh g^−1^ at 2 A g^−1^) and robust long-term stability (above 80 mAh g^−1^ after 2,000 cycles at 1 A g^−1^) at room temperature.
- DFT simulation verifies that the well-designed structure restricts FeS_2_ pulverization and facilitates the kinetic process of active ion.

**Supplementary Information:**

The online version contains supplementary material available at 10.1007/s40820-021-00682-8.

## Graphical abstract

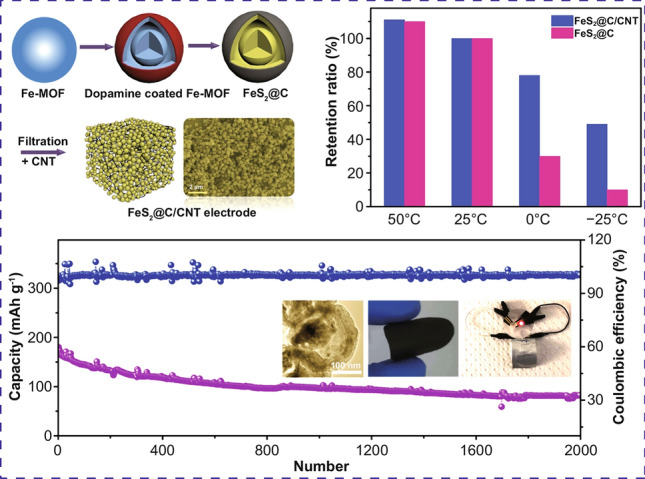

## Introduction

Rechargeable aluminum-ion batteries (AIBs) are promising next-generation batteries with merits of the most abundant metal resource on the earth crust, low cost, inherently safe handling, and the highest volumetric capacity (8.04 *vs.* 2.06 Ah cm^−3^ of lithium) [1, 2]. Previously, the intrinsic hydrogenation over the Al anode and passive oxide layer formation in the aqueous system drastically reduced the battery voltage and efficiency [3–8]; then, ionic liquid (IL) electrolytes were proposed to avoid these issues in the 2010s [1, 2, 9]. Since then, various cathodes have been proposed in IL-based AIBs including graphite-based materials, metal oxide/sulfide/selenium, MXene, and polymer-based materials to further improve the non-aqueous AIBs [10–15]. Yet, the IL-based AIBs still encountered several critical issues in terms of electrode material disintegration, short-term stability, and poor rate capacity (e.g., most metal sulfide cathodes have low rate capacity) toward practical applications [16, 17]. Therefore, it is highly desirable to construct new structured electrode materials with high capacity, long-term stability, and enhanced rate capability in the rechargeable AIBs.

Moreover, although the newly developed ILs have wide operating temperature window (e.g., from −50 to 80 °C), the development of the all-climate AIBs is severely hindered due to their inherently low capacity [18]. The emerging high-capacity metal sulfide electrodes are promising all-climate candidates, while their low ion/electron conductivity and inferior cycling stability need to be further optimized for all-climate AIBs [18–23]. FeS_2_, a earth-abundant (pyrite) and low-cost mineral with high theoretical capacity and favorable ion/electron conductivity, is a commercial cathode material in all-climate, especially under low temperatures, lithium batteries (such as Energizer L91) [24–28]. We were inspired to explore the use of FeS_2_ as an all-climate electrode in AIBs, which has not yet been reported.

Herein, we propose the design of a self-standing and binder-free carbon nanotube (CNT) wrapped metal–organic framework (MOF)-derived carbon-coated FeS_2_ (FeS_2_@C/CNT) as the high-capacity all-climate metal sulfide cathode in AIBs with exceptional flexibility. The binder-free and self-standing yolk–shell structure efficiently eliminates the side reaction between binder/current collector with IL electrolyte and tolerates volume expansion with robust cycling performance (above 80 mAh g^−1^ after 2,000 cycles at 1 A g^−1^). The density functional theory (DFT) simulation also verifies that the well-designed *N*-doped carbon shell not only restricts FeS_2_ pulverization but also facilitates the kinetic process of active ion toward FeS_2_@C/CNT, which is highly beneficial to the capacity/rate capacity (286 mAh g^−1^ at 100 mA g^−1^ and even 151 mAh g^−1^ at 2 A g^−1^). Moreover, the high conductive carbon matrix and porous structure significantly improve the electron/ion diffusion pathway and electrolyte infiltration with outstanding all-climate performance (−25 to 50 °C), which contribute to enhanced capacity retention (above 117 mAh g^−1^) and rate capacity even at a low temperature of −25 °C. The novel design of this FeS_2_@C/CNT paves the potential strategy toward high-performance all-climate and flexible AIBs.

## Experimental Section

### Chemicals

All chemicals were of analytical grade and used directly without further purification. Polyvinylpyrrolidone (PVP, K30), potassium ferricyanide (K_3_[Fe(CN)_6_]), hydrochloric acid (HCl, 36.0% ~ 38.0%), and dopamine hydrochloride were purchased from Sigma-Aldrich. Single-walled carbon nanotube (CNT, P3, > 90% purity) was obtained from carbon solution, Inc. All the reagents were used without further purification.

### Material Preparation

#### Yolk–shell Fe-MOF Spheres

PVP (3.00 g) and K_3_[Fe(CN)_6_]·3H_2_O (132 mg) were added to a 0.01 M HCl solution (40.0 mL) under magnetic stirring. After 30 min of stirring, a clear solution was obtained. The vial was then placed into an electric oven and heated at 80 °C for 24 h. After aging, the precipitates were collected by centrifugation and washed several times in distilled water and ethanol. After drying at room temperature for 12 h, Fe-MOF spheres were obtained. To obtain a yolk–shell structure, Fe-MOF (50 mg) and PVP (100 mg) were added to a 2.0 M HCl solution (30 mL) in a Teflon vessel under magnetic stirring. After 1 h, the vessel was transferred into a stainless autoclave and heated at 140 °C for 4 h. The etching time of 2 and 6 h were also conducted for comparison. After being cooled to room temperature, the precipitate was collected by centrifugation, then washed with deionized water and ethanol several times, and finally dried at 60 °C. Finally, yolk–shell Fe-MOF was obtained.

#### ***Yolk–shell FeS***_***2***_***@C Spheres***

First, 50 mg of yolk–shell Fe-MOF nanocubes and 25 mg of dopamine (DPA) were dispersed into a Tris-buffer solution (80 mL, 10 mM) with magnetic stirring for 3 h. The resultant product was collected via centrifugation and washed three times with deionized water and ethanol, respectively, and dried at 60 °C overnight. Then, the DPA-coated Fe-MOF (Fe-MOF@DPA) and sulfur powder were put at two separate positions in a porcelain boat with sulfur powder at the upstream side of the furnace. The weight ratio of Fe-MOF@DPA to sulfur is 1:5. After flushed with Ar, the center of the furnace was elevated to 500 °C at a ramping rate of 2 °C min^−1^, held at this temperature for 2 h, and then naturally cooled to ambient temperature under Ar.

### Fabrication of FeS_2_@C/CNT Electrode and AIBs

The flexible FeS_2_@C/CNT film was fabricated by the vacuum filtration method. Firstly, 15 mg of CNT and 35 mg FeS_2_@C were dispersed in 80 mL H_2_O using an intensive ultrasonication probe for 30 min. Then, the mixed solution was filtered through a mixed cellulose ester membrane (1.2 µm pore size, Millipore). The obtained filter cake was then vacuum-dried for 24 h to get a freestanding film.

The binder-free and freestanding FeS_2_@C/CNT directly utilized as a cathode in the AIBs. To compare with commercial FeS_2_, the FeS_2_ was firstly mixed with KB carbon with the same carbon content in FeS_2_@C/CNT. Then the mixture and polytetrafluoroethylene (PTFE) (weight ratio of 9:1) mixed in deionized water followed by overnight high-vacuum heating under 100 °C. The areal loading of the active materials is around 1.0 mg cm^−2^. The 1-ethyl-3-methylimidazolium chloride ([EMIm]Cl, 98%, Sigma) mixed with anhydrous aluminum chloride (99.99%, Sigma-Aldrich) (mole ratio of 1.3) to obtain the ionic liquid (IL) electrolyte (35 μL). A piece of glass fiber was used as the separator (Whatman). The aluminum foil (Sigma, 99.999%, 0.25 mm) is directly treated as the anode electrode. We applied soft package and Swagelok-type cells to assemble the batteries in glove box filled with Ar gas.

### Materials Characterization & Electrochemical Measurements

The samples were characterized by X-ray diffraction (XRD) (Bruker, Cu Kα, λ = 0.15406 nm, D8-Advance X-ray diffractometer,). The transmission electron microscopy (TEM), scanning electron microscopy (SEM) (JEOL-7001), and high-resolution TEM (HR-TEM) (FEI F20 FEG-STEM) were used to characterize the morphology of samples. The contact angles of ILs (drop on glass substrates under various temperature) were test via optical tensiometer (OCA 15 E/B). The electrochemical performance of the FeS_2_@C/CNT, and FeS_2_/C was tested by battery tester (LAND-CT2001A). The cyclic voltammogram (CV) was detected via electrochemical station (CHI 604e Shanghai, China) under a scan rate of 0.2 mV s^−1^.

### Computational Details

All the DFT calculations were performed with the Perdew–Burke–Ernzerhof (PBE) functional using the VASP code. The project-augmented wave (PAW) method was applied to represent the core–valence electron interaction [29]. The valence electronic states were expanded in plane wave basis sets with energy cutoff at 500 eV. For the bulk structure, 5 × 5 × 5, 5 × 5 × 3, 4 × 4 × 2 *k*-point mesh was used for cubic FeS_2_, hexagonal FeS, and hexagonal Al_2_S_3_. The convergence criterion of the total energy was set to be within 1 × 10^−5^ eV for the k-point integration and the force threshold for the optimization was 0.01 eV Å^−1^. The ion–electron interaction was described with the PAW method. A FeS_2_ (001) and graphene slab models were employed to simulate the surface properties. The Monkhorst–Pack method with the centered k-point grid (1 × 2 × 2) was used for surface calculations, respectively. All of the calculations were continued until the force has converged to less than 0.02 eV Å^−1^, and energies have converged within 10^–5^ eV.

The hydrogen and water adsorption energy on various surfaces is defined as Eq. (1):

1 $$\Delta E_{{{\text{ads}}}} = ~E_{{{\text{base}} - {\text{Al}}}} - ~E_{{{\text{base}}}} - E_{{{\text{Al}}}}$$

where $E_{{{\text{base}} - {\text{Al}}}}$ is the total energy of the slab model with Al adsorption, $E_{{{\text{base}}}}$ is the energy of a clean slab surface, and $E_{{{\text{Al}}}}$ is that for Al species, which refers to the Al single atom.

## Results and Discussion

### Synthesis and Characterizations of the FeS_2_@C/CNT

**Scheme 1:**
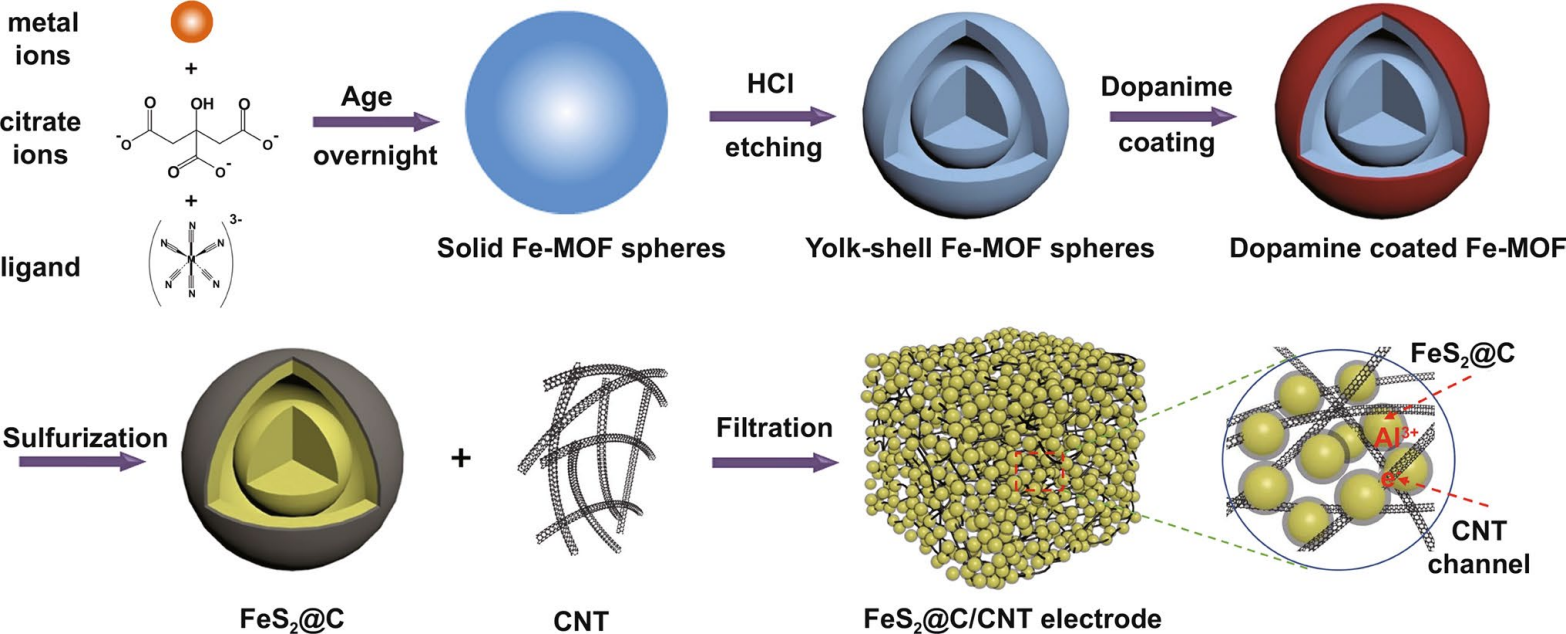
Schematic synthesis of FeS_2_@C yolk–shell spheres and self-standing/bind-free FeS_2_@C/CNT with an interpenetrative network structure

Scheme 1 presents the preparation of the yolk–shell spheres MOF-derived FeS_2_@C and the binder-free/self-standing FeS_2_@C/CNT film. The Fe-MOF was prepared via the hydrothermal method (a detailed synthetic condition in the supplementary materials). Then the Fe-MOF was treated in the acidic condition to obtain the yolk–shell hollow Fe-MOF spheres, which enabled abundant active sites to improve the electrolyte infiltration. The dopamine was coated and calcined to improve the electron conductivity of the electrode and control the volume expansion during the cycling. After sulfurization, the yolk–shell MOF-derived FeS_2_@C was obtained. Based on previous literature [12], the commonly used binders and current collectors have unexpected side reactions with Lewis acid IL electrolyte, which results in capacity decay and material pulverization. To avoid these issues, CNTs were simultaneously prepared with the FeS_2_@C to obtain binder-free and freestanding FeS_2_@C/CNT electrode.

**Fig. 1:**
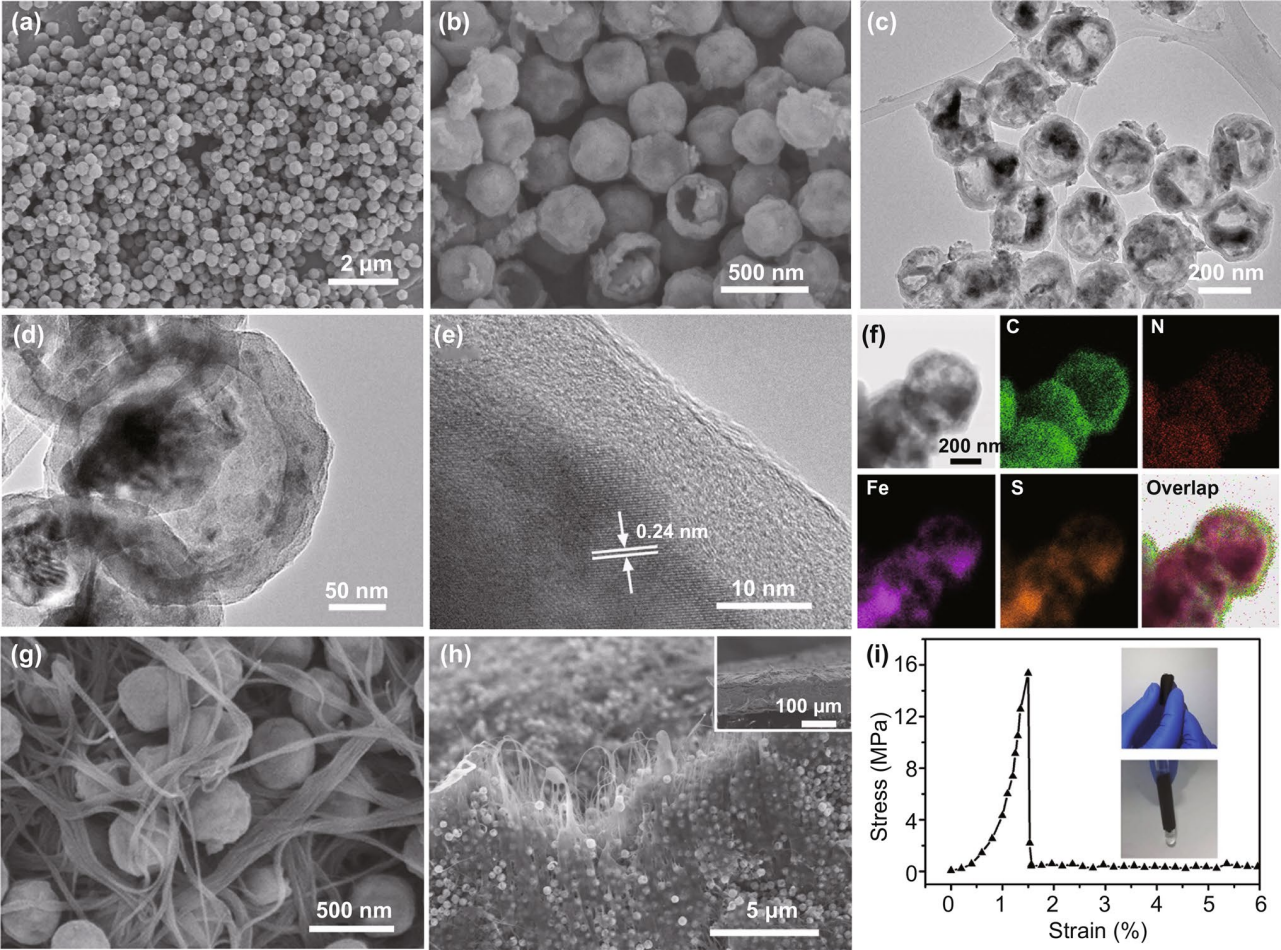
SEM images of **a** Fe-MOF, **b** FeS_2_@C yolk–shell nanospheres. **c, d** TEM and **e** HR-TEM images of yolk–shell FeS_2_@C. **f** Element mapping of the FeS_2_@C. **g, h** SEM images of the surface and fractured FeS_2_@C/CNT electrode. Inset in **h** exhibits the thickness of the FeS_2_@C/CNT. **i** Stress–strain curve of the flexible FeS_2_@C/CNT electrode with the folded electrode inset

Figure 1a displays the field emission scanning electron microscopy (FE-SEM) of the Fe-MOF. To obtain yolk–shell structure FeS_2_@C, the pristine solid nanospheres (Fe-MOF, Fig. S1) was etched in acid with different periods. The SEM and TEM images of the etched Fe-MOF (Figs. S2a, b) indicate the transformation from solid nanospheres to the hollow nanospheres after etching. The etching process were optimized via comparing with different conditions. More detailed morphological evolution under different etching stages is presented in Figs. S3 and S4, which was similar to the preparation of other MOF-derived materials [30–32]. As the etching time increases, the structure of the original Fe-MOF was gradually destroyed. After 2 h, a yolk–shell structure was formed based on the original Fe-MOF (Fig. S3). The cavity became larger after 4 h (Fig. S2), and each of the resultant samples possessed a spherical yolk–shell structure. After 6 h of etching, some of the particles were destroyed and a large interior hollow cavity was formed in the center of the Fe-MOF (Fig. S4). In order to maintain the complete and uniform structure of the material as well as reserve suitable space for the volume expansion of FeS_2_ sulfide during the cycling, 4 h of etching was selected as the optimal solution. Figure 1b shows the SEM image of dopamine-coated and sulfurized material (FeS_2_@C), which still maintains similar nanospheres morphology with the previous Fe-MOF. The TEM images of the FeS_2_@C (Figs. 1c, d) exhibit the yolk–shell hollow structure. The HR-TEM image shows a thin layer of carbon-coated on the surface of the crystal FeS_2_ (Fig. 1e). Moreover, a set of lattice fringes with *d*-spacing of 0.24 nm that are associated with the (210) plane of FeS_2_ crystals (JCPDS No. 42–1340) can be observed. The EDS mapping of the FeS_2_@C (Fig. 1f) not only indicates that Fe, S, N, and C elements are homogeneously distributed throughout the hollow yolk-sheath material but also confirms the nitrogen-doped sheath derived from the dopamine [12]. Based on the thermal gravimetric analysis data (Fig. S5), the mass content of carbon in FeS_2_@C is around 10 wt%. The XRD pattern of the product also proves that the obtained production with the characteristic peaks (Fig. S5) matches with the standard FeS_2_ phase (JCPDS No. 65–1765).

The binder-free and self-standing FeS_2_@C/CNT electrode is shown in the SEM image (Fig. 1g). The nanosphere FeS_2_@C was surrounded by the CNT substrate. The cross-view structure of the FeS_2_@C/CNT (Fig. 1h) also exhibits the homogenous distribution of the FeS_2_ particle among the CNT. The thickness of the FeS_2_@C/CNT, which can be controlled via modifying the added raw materials during the filtration process, is around 100 μm (inset of Fig. 1h). Moreover, the mechanical property of the FeS_2_@C/CNT is presented in the stress–strain curve (Fig. 1i). Maximum stretch stress (15 MPa) was achieved at the strain percentage of 1.4%. The considerable mechanical properties of the tested sample proved the enhanced flexible feature of this electrode.

### Electrochemical Performance of FeS_2_@C/CNT

**Fig. 2:**
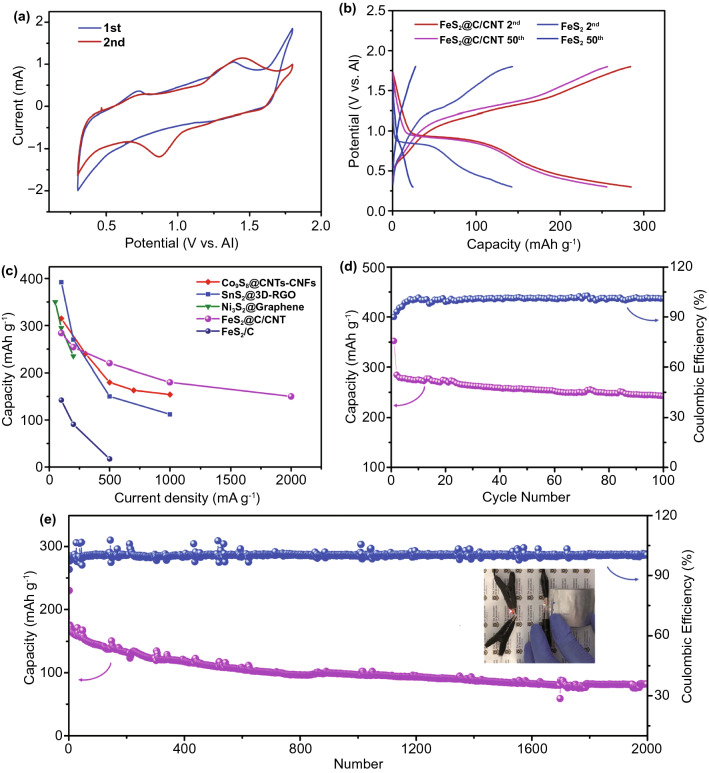
**a** 1st and 2nd cyclic voltammogram curves of FeS_2_@C/CNT. **b** 2nd and 50th galvanostatic discharge–charge curves of the FeS_2_@C/CNT and FeS_2_/C electrode at a current density of 100 mA g^−1^. **c** Rate capacity of FeS_2_@C/CNT and FeS_2_/C electrode in comparison with representative cathodes in AIBs [10–12]. **d** Cycling performance of the FeS_2_@C/CNT under a current density of 100 mA g^−1^. **e** Discharge capacity and Coulombic efficiency versus cycle number of FeS_2_@C/CNT electrode at a high current density of 1 A g^−1^. Inset of **e** exhibits the flexible FeS_2_@C/CNT in soft package AIBs

The electrochemical performances of the FeS_2_@C/CNT and normal FeS_2_ with carbon mixture electrode (FeS_2_/C) cathodes under room temperature are presented in Fig. 2. The cyclic voltammogram (CV) of the FeS_2_@C/CNT (Fig. 2a), which is conducted at 0.2 mV s^−1^, exhibits the initial cycle with weak peaks around 1.2 and 0.6 V during the anodic oxidation process and 0.7 and 1.3 V during the reduction process. Meanwhile, the initial discharge–charge curves (Fig. S6) also exhibit similar plateau during the initial discharge–charge process, which is assigned to the conversion of FeS_2_, accompanied by the formation of solid electrolyte interface (SEI) [10, 33]. The initial discharge capacity (as high as 352 mAh g^−1^) with irreversible capacity is ascribed to the SEI and side reaction between the electrolyte and electrode [33]. After the activation process in the first cycle, the discharge–charge curves display stable discharge plateau around 0.9 V and charge platform ca. 1.2 V (Fig. 2b), which are consistent with the corresponding oxidation and reduction peaks in the CV curves (2^nd^ cycle, Fig. 2a). To avoid the electrolyte decomposition at high voltage, the galvanostatic discharging–charging of the FeS_2_/C and FeS_2_@C/CNT was tested at a current density of 100 mA g^−1^ between 0.3 and 1.8 V. In Fig. 2b, the capacity of pristine FeS_2_/C cathode drastically reduces from 143 to 25 mAh g^−1^ after 50 cycles. Compared with the inferior capacity of FeS_2_/C and declined capacity after cycled, the discharge capacity of FeS_2_@C/CNT reached as high as 286 mAh g^−1^ and retained above 256 mAh g^−1^ after 50 cycles. The enhanced performance of FeS_2_@C/CNT was mainly ascribed to the carbon-coated structure, conductive matrix, and enhanced active sites of yolk–shell structure.

The rate capacity and cycling stability were also significant parameters for AIBs. The rate capacity of FeS_2_@C/CNT is compared with normal FeS_2_/C and other representative cathodes in AIBs (Fig. 2c) [10–12]. Even under a high current density of 2 A g^−1^, the FeS_2_@C/CNT could still deliver a capacity of 151 mAh g^−1^, which was among the best rate performance of AIBs reported to date [7]. Note that, due to the initially irreversible capacity, the listed capacity of FeS_2_@C/CNT were the capacity of second cycle. The superior rate capacity is mainly ascribed to the well-designed structure, high conductive nitrogen-doped carbon (N–C) layers, and CNT matrix. The coated N–C layer also highly restricted the expansion of the FeS_2_ and facilitated the kinetic process, which would be discussed in detail in the following part. Furthermore, the binder-free and freestanding feature would drastically reduce the side reaction between the electrolyte and binder/current collector [34]. In Fig. 2d, the FeS_2_@C/CNT maintains a high capacity of 241 mAh g^−1^ after 100 cycles under a current density of 100 mA g^−1^. Even under a high current density of 1 A g^−1^, the FeS_2_@C/CNT exhibits above 80 mAh g^−1^ discharge capacity after long-term cycling of 2,000 times with above 95% Coulombic efficiency (Fig. 2e). Some unstable cycles were mainly ascribed to the temperature during the long-term testing. The flexible AIBs based on the FeS_2_@C/CNT (two batteries back to back as shown in the inset of Fig. 2e) can light up an LED, indicating the promising potential of FeS_2_@C/CNT in flexible AIBs.

### Mechanism and DFT Investigation

**Fig. 3:**
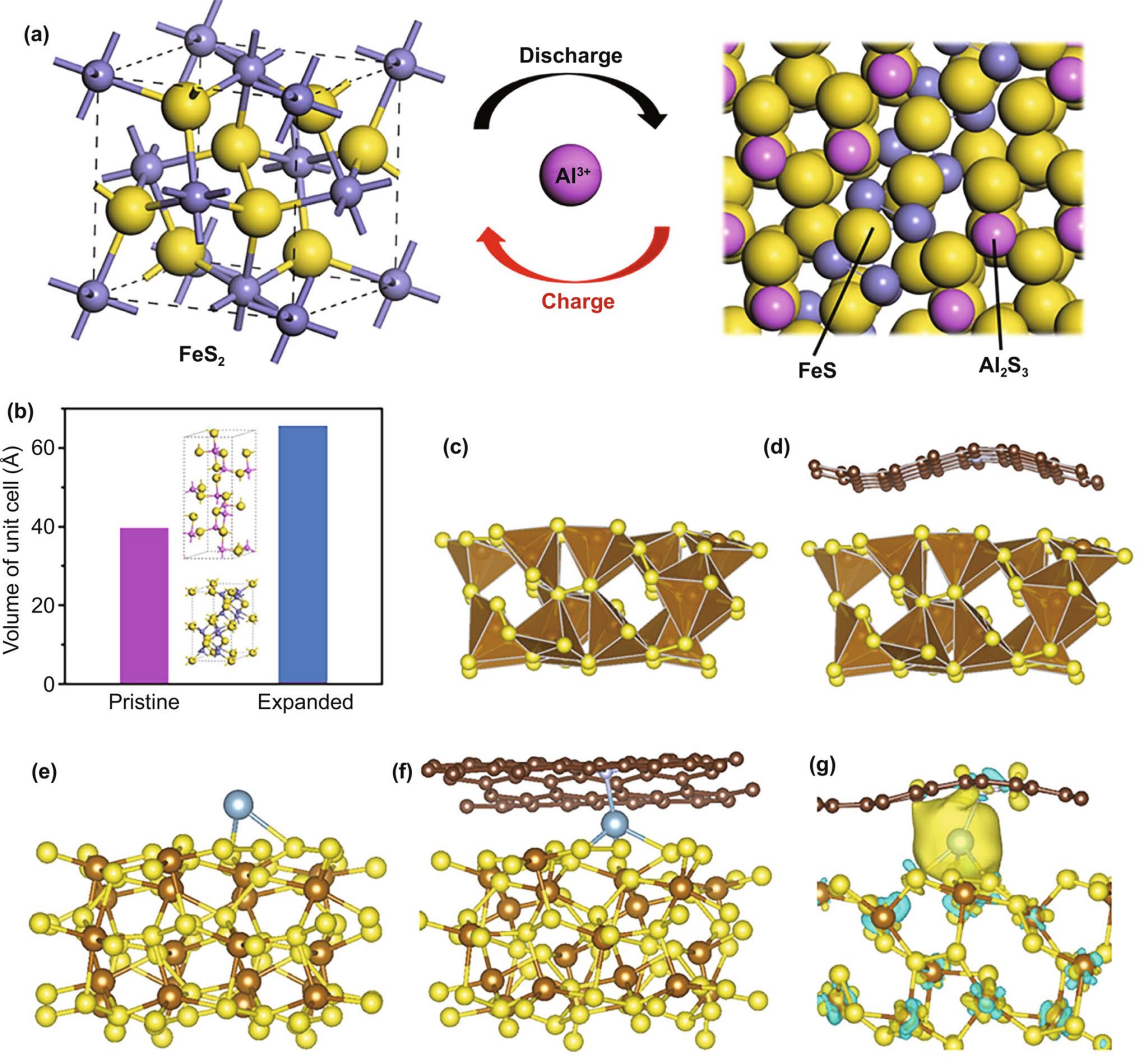
**a** Schematic process of the charge–discharge process toward AIBs. **b** Simulation of the volume expansion of the FeS_2_ in a unit cell. **c-d** Feasible models for optimal FeS_2_ and adsorption sites of FeS_2_ on N–C. **e–f** Feasible models for optimal adsorption sites of Al ion on FeS_2_ and between the N–C and FeS_2_. **g** Different charge density of Al ion between N–C and the FeS_2_, the isosurface value is set to be 0.05 e Å^−3^. The blonde, brown, nigger brown, and blue balls represent S, Fe, C, and Al atoms

X-ray photoelectron spectroscopy (XPS), TEM, and DFT calculations were further applied to investigate the reaction process and explore the origin of the electrochemical feature of FeS_2_@C/CNT. The electrochemical process of FeS_2_ was characterized via XPS and then simulated via DFT calculations. In Fig. S7, the 2p spectra of S with two major doublet peaks at 162.7 (2p_3/2_) and 163.8 (2p_1/2_) eV can be signed to the S_2_^2−^ in pristine FeS_2_ [35]. After discharged, the 2p spectra of S shifted to the low binding energy with peaks at 161.7 (2p_3/2_) and 163.4 (2p_1/2_) eV which could be signed to the S^2−^ in FeS [36]. After charged, the single shifted back to the high binding energy at 162.7 (2p_3/2_) and 163.9 (2p_1/2_) eV, which also indicates the reversibility of the reaction. The result of the conversion reaction between FeS_2_ and FeS is consistent with the previous report on FeS_2_ in AIBs [37]. Meanwhile, the variation of XPS spectra of Fe 2p during the discharge–charge process is similar to the reported variation from FeS_2_ to FeS (Fig. S7), which further confirms the proposed conversion reaction of the FeS_2_. The simulation of the electrochemical process of FeS_2_ is conducted via the DFT calculation (Fig. 3a). Based on the simulation, the electrochemical process would generate a drastically volumetric expansion of active material. The unit cell of the FeS_2_ (39.48 Å^3^) increased to a large volume (65.52 Å^3^) upon the discharge process. The detailed simulation result of the involved materials is listed in Table S1. The above 160% volume expansion in the unit cell will generate serious electrode material pulverization and irreversible capacity with inferior cycling stability (Fig. 3b), which results in the inferior performance of the normal FeS_2_/C electrode.

To explore the origin of the electrochemical feature of the FeS_2_@C/CNT electrode, particularly the cycling stability and rate capacity, SEM, TEM, and DFT were utilized to investigate the designed electrode during the electrochemical process. Based on the above simulation result of volumetric variation during the electrochemical process, a feasible strategy was required to accommodate the volume expansion of active materials [38]. The FeS_2_@C/CNT owns not only the yolk–shell structure but also extra void space (Fig. 1d). Thus, this yolk–shell structure with the outermost carbon layer restrained the expansion of the active material and stabilized the electrode. The void space inside was sufficient to tolerate the volume variation, which was also beneficial to the electrode stability. Figure 3c, d shows the optimal FeS_2_ and the loading of FeS_2_ on the N-doped carbon, which is corresponding to the N-doped carbon layer in FeS_2_@C/CNT models. The obvious folded structure of the N–C layer revealed the strong interaction between FeS_2_ and N–C, which also effectively reduced the FeS_2_ splitting up from the carbon structure and active material pulverization. Furthermore, the morphology of FeS_2_@C/CNT after 50^th^ and 200^th^ cycles (Fig. S8) are slightly cracked compared with the initial sample (Fig. 1d), which confirms the simulation results on this stable yolk–shell FeS_2_@C/CNT. On the contrary, the micro-FeS_2_ in the normal FeS_2_/C electrode is seriously degenerated after cycling with obvious cracking and pulverization (Figs. S9 and S10).

**Fig. 4:**
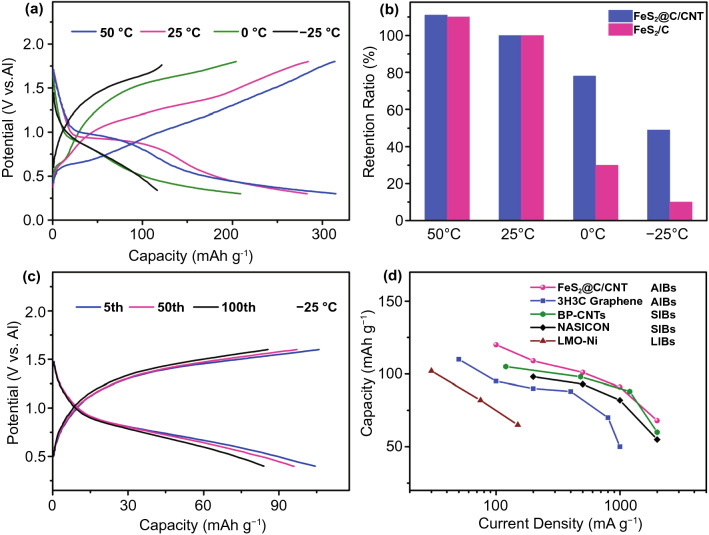
**a** Discharge–charge curves of the FeS_2_@C/CNT under a range of temperatures at a current density of 100 mA g^−1^. **b** Capacity retention of FeS_2_@C/CNT and FeS_2_/C electrodes at a current density of 100 mA g^−1^ from −25 to 50 °C. **c** 5^th^, 50^th^, and 100^th^ charge–discharge curves of the FeS_2_@C/CNT under −25 °C at a current density of 100 mA g^−1^. **d** Rate capacity of FeS_2_@C/CNT in AIBs and other representative low-temperature electrode in AIBs, SIBs, and LIBs at low temperature (~ −25 °C) [18, 39–41]

To better understand the kinetic process during the electrochemical reaction and enhanced rate capacity, the simulation of absorption of aluminum ion on pristine FeS_2_ and FeS_2_@N–C/CNT was conducted to reveal the advantage of the designed structure. Based on the simulation result (Figs. 3e, f), the aluminum ion between the N–C layer and FeS_2_ has much strong adsorption energy (−1.76 eV) in comparison with the bare FeS_2_ module (−1.41 eV) as shown in Table S2. The lower adsorption energy indicates the strengthened reaction process when Al ion involved, which is beneficial for the rate capacity of FeS_2_@N–C/CNT. Meanwhile, in Fig. 4g, when the Al locates between FeS_2_ and N–C, the charge accumulation (yellow ball) can be observed, which illustrates the obvious electron transfer within Al and FeS_2_/N–C and further proves the strong interaction between them. Based on the above characterization and simulation, the well-designed FeS_2_@C/CNT not only prevents the agglomeration of FeS_2_ particles during cycling but also accelerates the reaction process, contributing to the robust cycling and high rate capacity.

### All-climate Performance of FeS_2_@C/CNT in AIBs

Most of the studies on AIBs to date focus on room temperature or high-temperature electrochemical performance [37, 42]. Although the graphite-based AIBs have a considerable process under a wide temperature range, the inherently limited capacity (less than 150 mAh g^−1^ even at high temperature) still hinders the development of all-climate AIB, especially under low temperature [8]. Thus, emerging high-capacity metal chalcogenide-based electrodes, such as metal sulfides, were worth exploring, yet few reported. Based on the inherent ion-conductivity of FeS_2_, enhanced performance at room temperature, and above simulation of the kinetic process of the FeS_2_@C/CNT cathode, the battery performance of FeS_2_@C/CNT under all-climate, especially for the cold climate, was further investigated in detail. The inherent advantage of the IL-based AIBs contributed to enhanced electrochemical performance under low temperature. The contact angle of IL only increased from 55 to 64° when the temperature reduced from 25 to −25 °C (Figs. S11a-c). On the contrary, the considerable organic solvent-based electrolyte of LIBs and SIBs froze at −25 °C [43]. The wettability experiment of IL indicates that the stable infiltration of the IL under a range of temperatures from subzero to room temperature. Moreover, the IL electrolyte also shows stable ion conductivity under a range of temperatures (Fig. S11d). In Fig. 3a, the discharge–charge curves of the FeS_2_@C/CNT are exhibited under the temperature from −25 to 50 °C. Although the previous literature has reported the inferior cycling stability of batteries at high temperature [39], the FeS_2_@C/CNT exhibits stable capacity and Coulombic efficiency at 50 °C (Fig. S12), which is ascribed to the void yolk–shell structure and well-protected carbon layer to prevent the materials pulverization and side reaction. Owing to the reduced ion conductivity of the electrolyte and the inherently kinetic process on cathode/electrolyte under low temperature [40], the battery performance of FeS_2_/C drastically reduced under low temperature. However, the FeS_2_@C/CNT exhibit comparable high capacity retention even under −25 °C (above 117 mAh g^−1^ at 100 mA g^−1^, Figs. 4a, b). When compared with that of normal FeS_2_/C (only 10% capacity retention under −25 °C), the FeS_2_@C/CNT exhibited much higher capacity retention (above four times higher than that of FeS_2_/C). Although low temperature with slow kinetic process reduced the stability of the discharge–charge plateau, the MOF-derived carbon on FeS_2_, CNT matrix and hollow structure still largely enhanced the high conductivity and kinetic process of FeS_2_@C/CNT in compared with FeS_2_/C. Furthermore, the cycling stability of the FeS_2_@C/CNT under low temperature still maintains stable with around 85 mAh g^−1^ after the 100^th^ cycle at 100 mA g^−1^ (Fig. 4c). These features and improvements are ascribed to the high conductivity carbon matrix, hierarchical porous structure, and, more importantly, enhanced kinetic process as simulated above. The representative all-climate electrode, such as graphite-based AIBs, Prussian blue/CNTs (BP-CNTs) in SIBs, optimized NASICON in SIBs, and LiMn_2_O_4_ doped with Ni in LIBs were compared with the FeS_2_@C/CNT at a range of current densities under subzero temperature ~ −25 °C [18, 39–41]. The FeS_2_@C/CNT exhibited higher capacity performance among the representative low-temperature batteries (Fig. 4d). The inherently high ionic conductivity of IL under a range of temperatures (Fig. S11d) and the rational electrode design of the FeS_2_@C/CNT both contribute to the enhanced all-climate performance of FeS_2_@C/CNT.

## Conclusion

In summary, we prepared a free-standing and binder-free FeS_2_@C/CNT and applied in all-climate AIBs for the first time. The optimized electrode exhibited a high capacity (286 mAh g^−1^ at 100 mA g^−1^), robust cycling stability (above 80 mAh g^−1^ at 1 A g^−1^ after 2,000 cycles), remarkable rate performance (151 mAh g^−1^ under a high current density of 2 A g^−1^), and more importantly, excellent all-climate behavior (delivering above 117 mAh g^−1^ at 100 mA g^−1^ under low temperature of −25 °C). Based on the detailed characterization and DFT simulation, we reveal that the excellent electrochemical performance is ascribed to two main reasons; the yolk–shell structure with sufficient void space restricts volume expansion and electrode pulverization with robust electrode stability. The deliberately designed hierarchical structure and N–C-coated layer with CNT matrix not only allows abundant exposed active sites but also facilitates the kinetic process with improved ion/electron conductivity even under a wide temperature window. Furthermore, the binder-free and freestanding features reduce the active material disintegration and side reaction with significantly increased electrochemical stability under ambient temperature. The findings reported herein provide new insights into the rational design of high-performance composite electrodes for scalable and flexible AIBs in all-climate applications.

## Supplementary Information

Below is the link to the electronic supplementary material.

Supplementary file1 (PDF 1617 KB)
