# Supplementary material for: All-Climate Aluminum-Ion Batteries Based on Binder-Free MOF-Derived FeS2@C/CNT Cathode
Source: Nanomicro Lett. 2021 Jul 23;13:159. doi: 10.1007/s40820-021-00682-8 (PMC8302704; doi:10.1007/s40820-021-00682-8)
Supplement: Supplementary file 1 — Supplementary file1 (PDF 1617 KB) [file 40820_2021_682_MOESM1_ESM.pdf]

Supporting Information for

All-Climate Aluminum-Ion Batteries Based on Binder-Free MOF-Derived FeS_2@C/CNT Cathode

Yuxiang Hu[1], Hongjiao Huang[2], Deshuang Yu[2], Xinyi Wang[1], Linlin Li[2], Han Hu[3], Xiaobo Zhu[1], Shengjie Peng[2, *], Lianzhou Wang[1, *]

[1] Nanomaterials Centre, School of Chemical Engineering and Australian Institute for Bioengineering and Nanotechnology, The University of Queensland, QLD 4072, Australia

[2] Jiangsu Key Laboratory of Electrochemical Energy Storage Technologies, College of Materials Science and Technology, Nanjing University of Aeronautics and Astronautics, Nanjing, 211106 P. R. China

[3] State Key Laboratory of Heavy Oil Processing, College of Chemical Engineering, P. R. China University of Petroleum (East China), Qingdao 266580, P. R. China

*Corresponding authors. E-mail: l.wang@uq.edu.au (Lianzhou Wang); pengshengjie@nuaa.edu.cn (Shengjie Peng)

Supplementary Figures and Tables

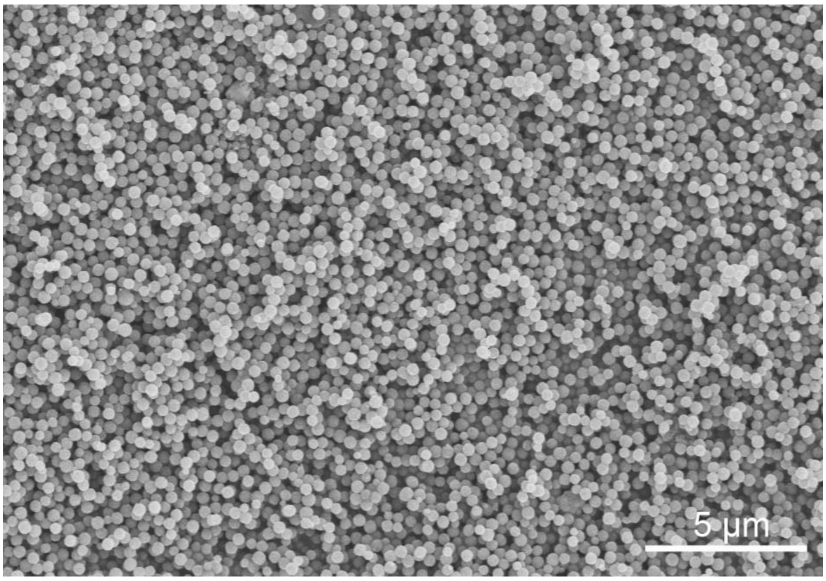

Fig. S1 SEM image of Fe-MOF on a large scale

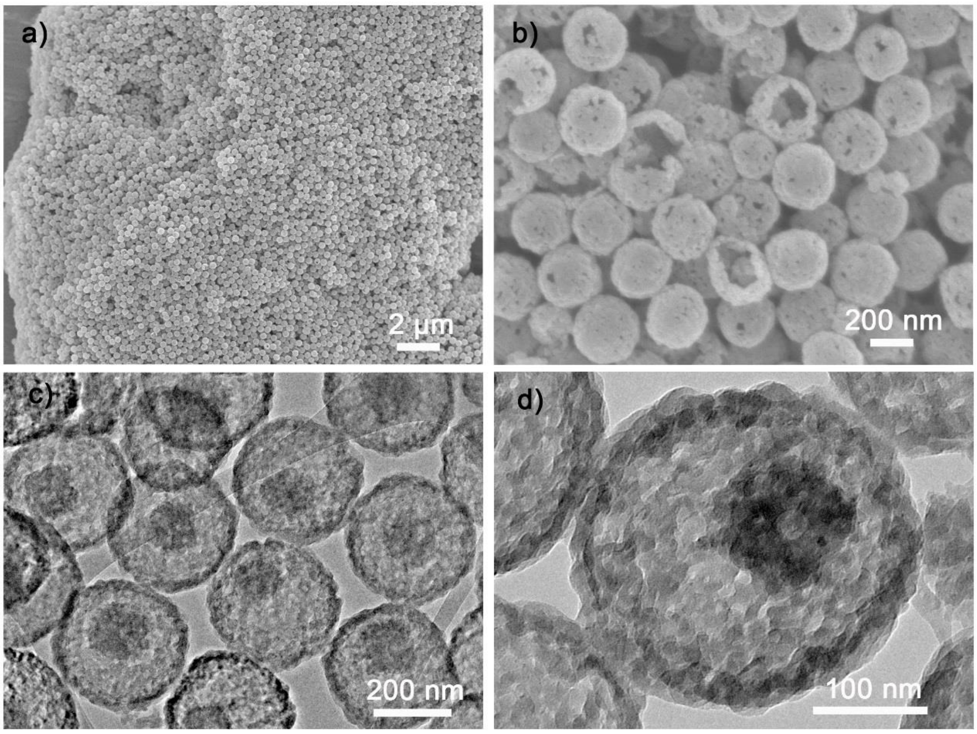

Fig. S2 a), **b**) SEM and **c**), **d**) TEM images of the etched Fe-MOF with yolk-shell structure

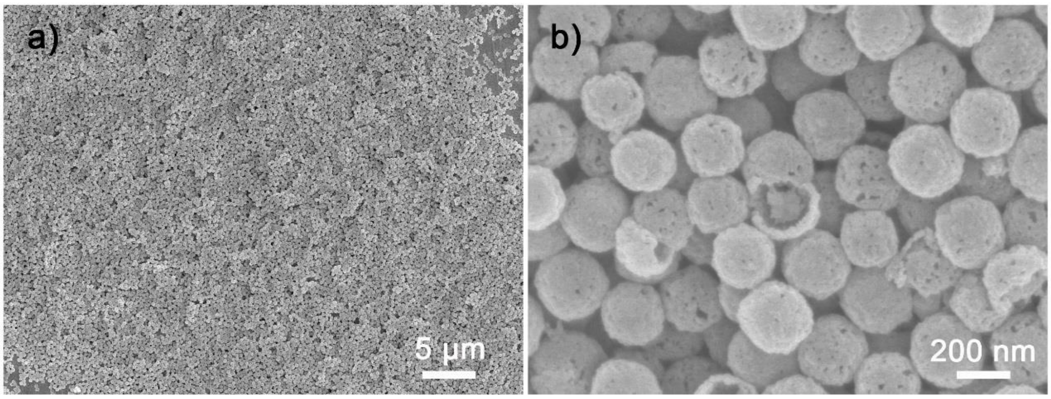

Fig. S3 SEM image of Fe-MOF after etching by acid at 140 °C for 2 h

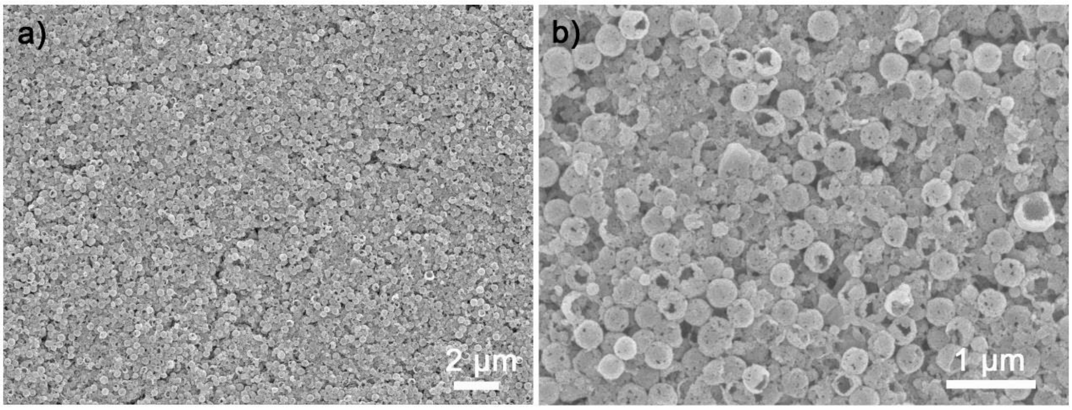

Fig. S4 SEM image of Fe-MOF after etching by acid at 140 °C for 6 h

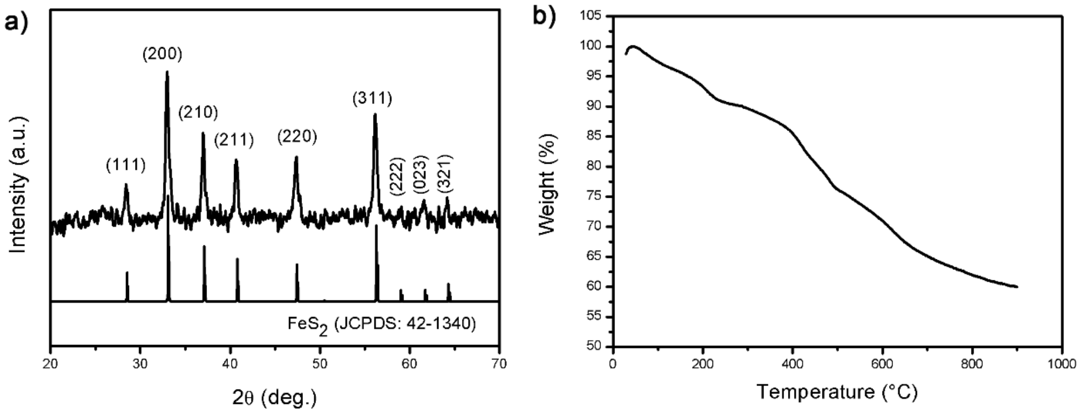

Fig. S5 a) XRD pattern and **b**) TGA curve of FeS_2@C. The TGA curve, which evaluates the weight of FeS_2 in FeS_2@C, was measured over a temperature range from 30 °C to 900 °C in an atmosphere of air. It can be observed that the weight loss is around 40%. The weight loss is ascribed to two reactions: carbon to CO_2 (reaction 1) and FeS_2 oxidized to Fe_3O_4 (reaction 2). Based on the weight loss in TGA result and following Eq. (S1) (where *M* indicates molecular weight, and *m* represents the mass of the samples), it can be calculated that the carbon weight is about 10 *wt*% in FeS_2@C.

$$C+O_2=CO_2 \qquad \text{reaction (1)}$$

$$3FeS_2+8O_2=Fe_2O_3+6SO_2 \qquad \text{reaction (2)}$$

$$FeS_2(wt\%)=100\times(3M_{FeS2}/M_{Fe2O3})\times(m_{Fe2O3}/m_{FeS2@C}) \qquad \text{(S1)}$$

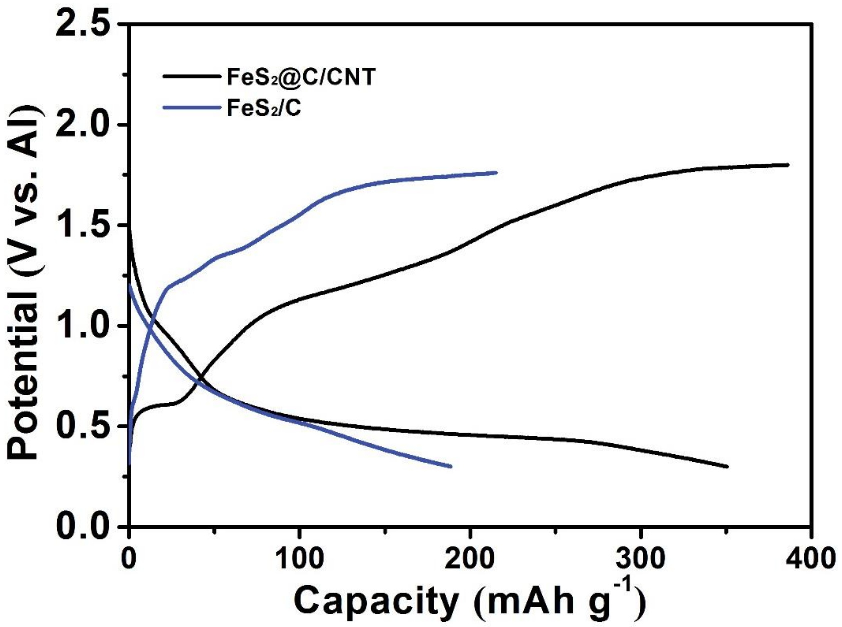

Fig. S6 Initial discharge-charge curves of the FeS_2@C/CNT and FeS_2/C electrode

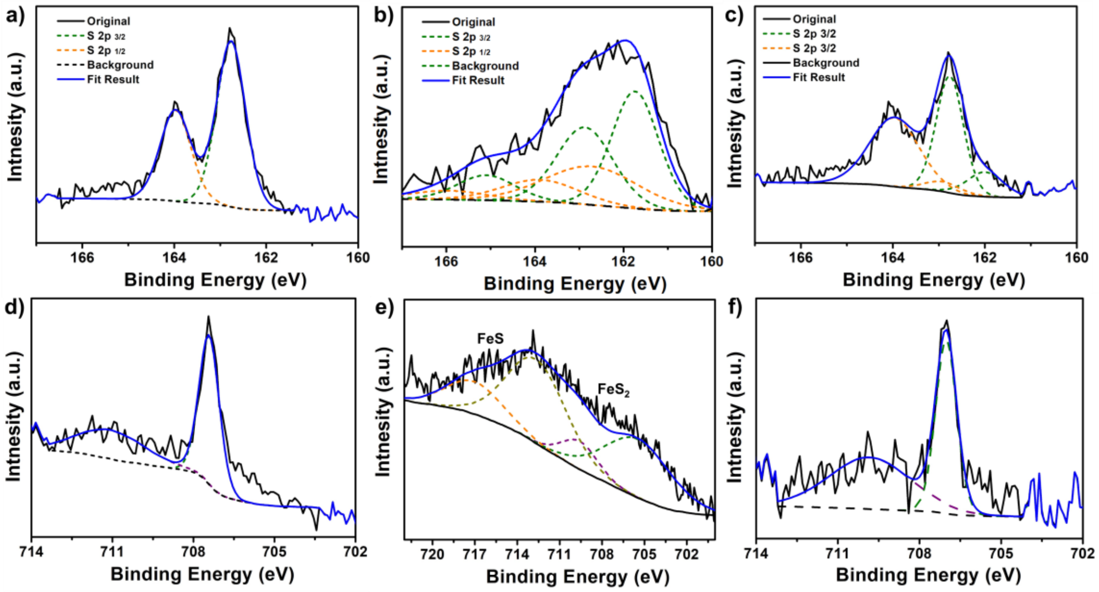

Fig. S7 XPS spectra of S 2p of the FeS_2 electrode at **a**) pristine, **b**) discharged, and **c**) charged stage. XPS spectra of Fe 2p of FeS_2 electrode at d) pristine, **e**) discharged, and **f**) charged stage. After fully discharged, the peak shift to the lower binding energy with the peak around 160.5 eV, which is ascribed to the S^{2-} in FeS. Meanwhile, there still existed remaining peaks contributed by S_2^{2-} from FeS_2 due to the inactive materials in the cathode. After fully recharged, the peaks shifted back the higher binding energy ascribing to the S_2^{2-} of FeS_2, which further proved the proposed reaction. Regards to the Fe 2p spectra, the postive shift of the peaks confirmed the formation of the FeS after fully discharged. The recovery of the Fe 2p peak after fully recharged further proved the reversibility of the proposed reaction.

Table S1 Simulation result of the involved materials during the discharge-charge process

	a/Å	b/Å	c/Å	α/degrees	β/v	γ/ degrees	V/Å^3
Fe_4S_8	5.405215	5.405215	5.405215	90	90	90	157.921
$Fe_{12}S_{12}$	6.019331	6.019331	11.16354	90	90	120	350.291
$Al_{12}S_{18}$	6.610146	6.610146	17.28143	90	90	120	653.932

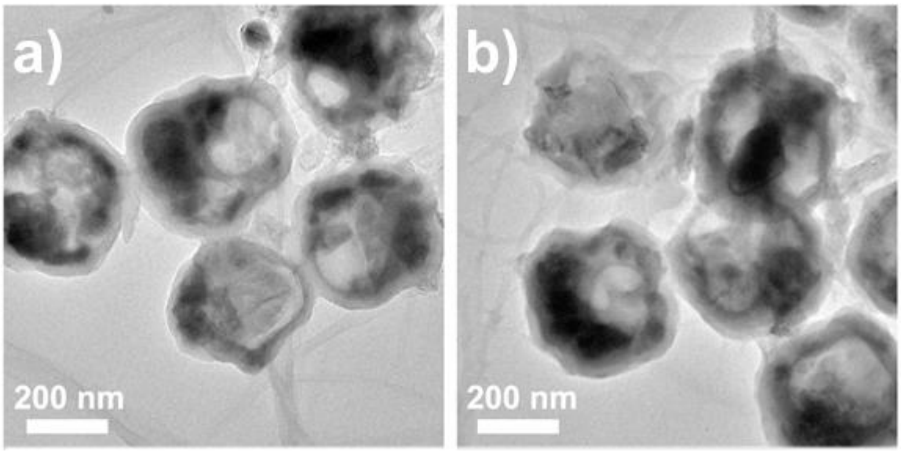

Fig. S8 a) and **b**) TEM image of FeS_2@C/CNT electrode after 50th and 200th cycles, respectively

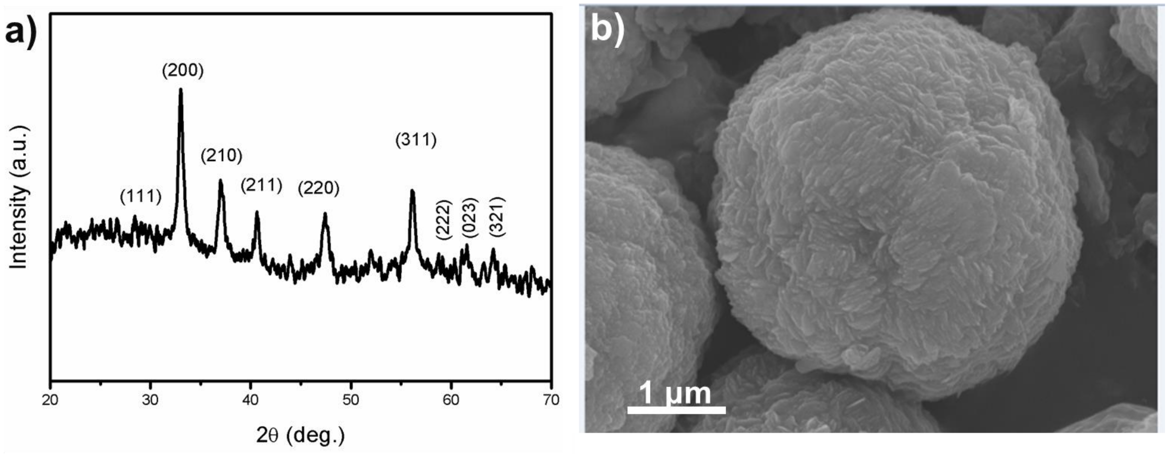

Fig. S9 a) XRD and **b**) SEM image of micro-sphere FeS_2 bulk, respectively

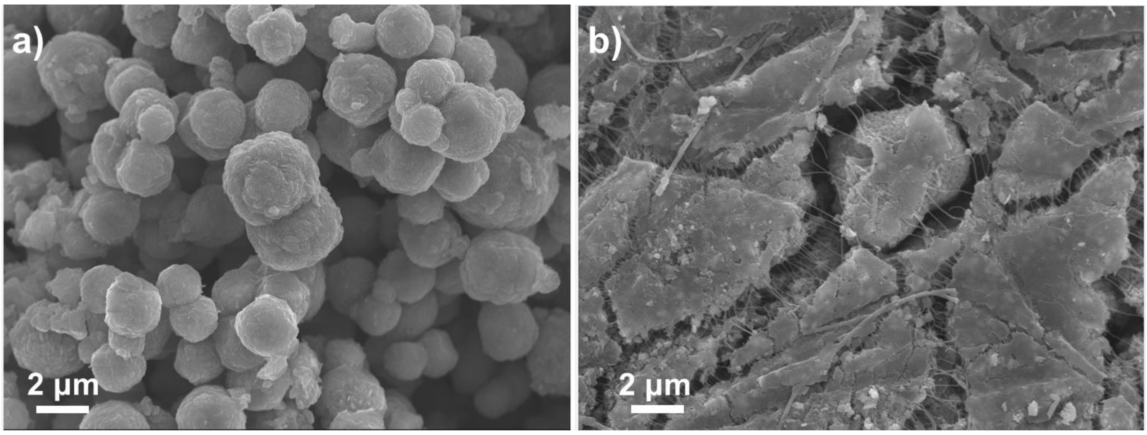

Fig. S10 SEM image of the micro-sphere FeS_2 **a**) before cycled and **b**) after cycled for 50 times

Table S2 Simulation result of the adsorption energy of the FeS_2 and FeS_2/N-C models

	FeS_2/N-C	FeS_2
E_{base}	-798.492	-369.06
E_{Al}	-0.197	-0.197
$E_{base} + E_{Al}$	-800.446	-370.668
ΔE	-1.755	-1.406

The hydrogen and water adsorption energy on various surfaces is defined as Eq. (S2):

$$\Delta E_{ads} = E_{base-Al} - E_{base} - E_{Al} \qquad \text{(S2)}$$

where $E_{base-Al}$ is the total energy of the slab model with Al adsorption, E_{base} is the energy of a clean slab surface, and E_{Al} is that for Al species.

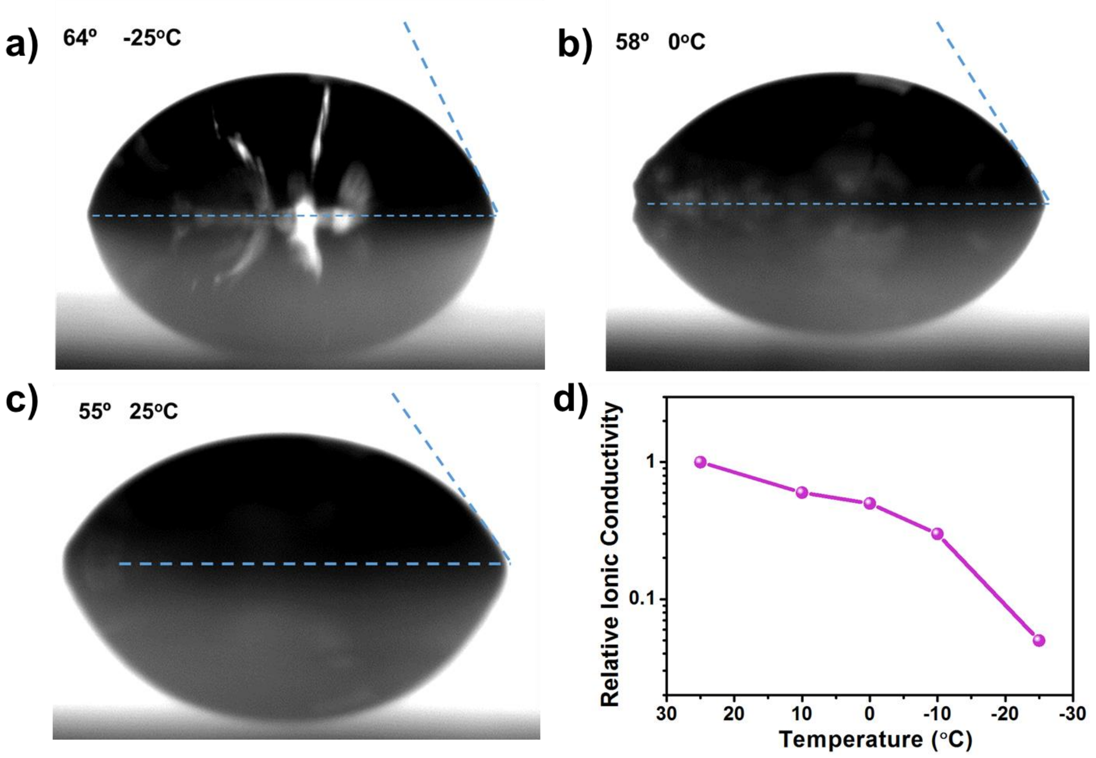

Fig. S11 Contact angle of the IL under **a**) -25°C, **b**) 0 °C, and **c**) 25 °C. **d**) the relative ionic conductivity of the IL electrolyte under various temperatures

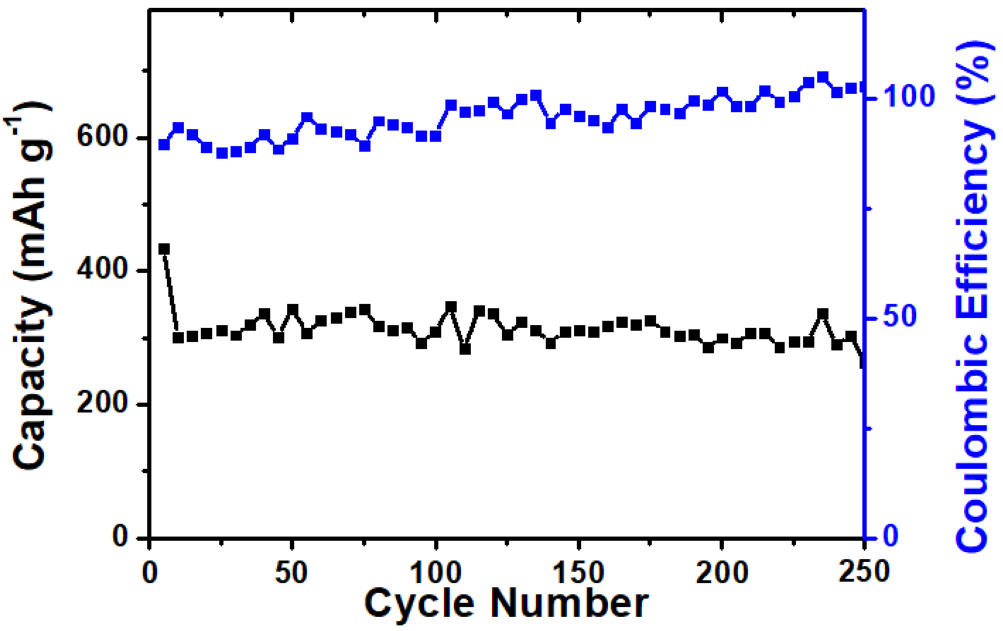

Fig. S12 Cycling stability of the FeS_2@C/CNT at a high temperature of 50 ºC. Although previous literature reported the inferior cycling stability of batteries at high temperature, the FeS_2@C/CNT maintained stable capacity and coulombic efficiency at 50 ºC, which was ascribed to the void yolk-shell structure and well-protected carbon layer. Meanwhile, the interaction between FeS_2 and N-doped carbon was discussed in the simulation part.
